# MIR548P and TRAV39 Are Potential Indicators of Tumor Microenvironment and Novel Prognostic Biomarkers of Esophageal Squamous Cell Carcinoma

**DOI:** 10.1155/2022/3152114

**Published:** 2022-09-17

**Authors:** Jian Xu, Long Tang, Zhiqiang Wang, Qi Zhang, Yuequan Jiang

**Affiliations:** Chongqing Key Laboratory of Translational Research for Cancer Metastasis and Individualized Treatment, Chongqing University Cancer Hospital, Chongqing 400030, China

## Abstract

Esophageal squamous cell carcinoma (ESCC) remains a common aggressive malignancy in the world. Multiple studies have shown evidence to support the hypothesis that certain functional genes that are engaged in the microenvironment of tumors played a role in the progression of ESCC. Thus, to better analyze the prognostic values of important genes in ESCC, there is an immediate need for an in-depth research study. From the TCGA database, the RNA-seq data and clinical features of 163 ESCC patients were obtained. Using the ESTIMATE technique, we were able to calculate the ImmuneScore, the StromalScore, and the ESTIMATEScore for each ESCC sample. The samples from the ESCC were split up into high score and low score groups based on the median of the various scores. In this study, ImmuneScore, StromalScore, and ESTIMATEScore were not found to be linked with overall survival of ESCC patients, according to our findings. Higher StromalScores were linked to more advanced T stages and clinical stages. The intersection analysis that was exhibited by the use of a Venn diagram indicated that there was a total of 944 upregulated genes that shared the same high score in both the ImmuneScore and the StromalScore and that there was 0 downregulated gene that shared the same low score. Survival experiments confirmed MIR548P and TRAV39 as critical prognostic biomarkers for ESCC patients. Importantly, we found that TRAV39 expression was positively associated with T cell CD4 memory activated while negatively associated with B cell memory, dendritic cells activated, and mast cells activated. In addition, we found that MIR548P expression was negatively associated with mast cells activated while positively associated with T cell CD4 memory activated. Overall, we identified MIR548P and TRAV39 as new modulators for ESCC, affecting the immune microenvironment of ESCC patients and may be a target of immunotherapy.

## 1. Introduction

Esophageal cancer has become a common malignant tumor in this world [1]. In addition, it is a significant contributor to deaths caused by cancer [2]. The number of cases of esophageal cancer, which are sadly rising at an alarming rate, will not stop rising [3]. Esophageal squamous cell carcinoma (ESCC) is the predominant histological type of esophageal cancer worldwide [4]. Due to the absence of typical symptoms in the early stage, patients who have ESCC are always detected at a late stage in the disease's progression [5, 6]. On the other hand, metastasis is one of the primary causes that leads to recurrence following surgical therapy, which ultimately results in the failure of the therapeutic attempt [7]. If the progression of the disease can be forecasted based on the identification of pertinent signs in patients, then the clinical prognosis of those patients will significantly improve [8, 9]. Although relevant immunotherapies involving ESCC are still in the basic stages of development, certain related immunosuppressants have been applied in specific patients and demonstrated long-lasting anticancer effectiveness as well as controlled adverse responses [10, 11]. The ability to make an accurate prognostic assessment of ESCC is essential to the efficacy of clinical screen and treatments, as well as customized medicine. Therefore, it is highly vital to identify unique and reliable prognostic biomarkers from different dimensions in order to determine the best appropriate therapy plans and improve the dismal outcome of patients with ESCC.

The beginning, the course, and the development of ESCC are all determined by the genes that are inherent to the tumor cells, particularly the master transcription factors [12, 13]. On the other hand, it has been observed that the microenvironment of the tumor has a significant impact on the gene expressions of the tumor specimens and, as a result, the long-term survivals [14, 15]. The microenvironment of tumor refers to the cellular milieu in which the tumor itself is situated. Inflammatory mediators and extracellular matrix (ECM) molecules are also a part of it, along with immune cells, endothelial cells, and mesenchymal cells [16, 17]. In the microenvironment of tumor, immune and stromal cells are two major types of nontumor components that could be useful for the diagnosis of tumors [18, 19]. Immune cells can help diagnose cancer, while stromal cells help predict how aggressive a tumor will become. It has been suggested that tumor-infiltrating immune cells (TIICs) and stromal cells, which are two important categories of nontumor cell components, are useful in the prediction of clinical outcome of malignancies [20, 21]. Previous researches have shown evidence that tumor-infiltrating lymphocytes (TILs) play a key role in determining the clinical progression of a variety of malignancies [22, 23]. Recently, several types of cancers, including renal, prostate, colorectal, ovarian, bladder, and lung cancers, have been linked to TILs, specifically cytotoxic T cells, memory T cells, and T helper 1 cells, which are positively related to good clinical outcomes [24, 25]. In addition to this, it was observed that the tumor microenvironment (TME) had an effect on the gene expression in the cancer specimens as well as the prognostic results. These findings shed light on the connection between the tumor microenvironment and the evolution of cancer, suggesting new approaches that could make the management of cancerous tumors more effective.

Through the use of the ESTIMATE algorithm, we were able to acquire the ImmuneScore and StromalScore of ESCC patients that were stored in the TCGA database. The purpose of this work was to determine which genes with key functional roles were implicated in TME. After that, we further explored their clinical significance.

## 2. Methods

### 2.1. Datasets and Data Processing

The TCGA-ESCA RNA-seq FPKM data, together with clinical data and survival data, were retrieved from the UCSC Xena database. There were 163 cases with ESCA and 11 normal cases, all of which had their clinical data extracted from the above datasets.

### 2.2. Generation of ImmuneScore, StromalScore, and ESTIMATEScore

Each sample's ratio of immune-stromal component in TME was estimated using the ESTIMATE algorithm implemented in R language version 3.5.1 with the estimate package and displayed as one of three scores: ImmuneScore, StromalScore, or ESTIMATEScore. ImmuneScore, StromalScore, and ESTIMATEScore all showed positive correlations with the ratio of immune, stromal, and the sum of the two components in TME, so higher scores indicated larger ratios of the respective component.

### 2.3. Distinguishing of Differentially Expressed Genes (DEGs)

The “limma” algorithm was used to perform preprocessing on the raw data that TCGA collected. The cut-offs for identifying DEGs were determined to be adjusted *p* values (adj. *p*) less than 0.05 and |Log2 (FC)| greater than 1.

### 2.4. Heatmaps and Clustering Analysis

The web application “ClustVis” was utilized in order to produce heatmaps [26].

### 2.5. Enrichment Assays of DEGs

R 4.0.2 and the related R packages were utilized to carry out Gene Ontology (GO) and the Kyoto Encyclopedia of Genes and Genomes (KEGG) enrichment assays, and the DEGs were utilized as the data source. Only terms whose *p* and *q* values were lower than 0.05 were judged to have significantly increased abundance.

### 2.6. TME Component-Related Survival Analysis

After performing survival analysis on all of the ESCC samples, we separated them into two groups, one with high scores and one with low scores. The Kaplan-Meier methods were applied to generate the survival curve, and the log-rank test was performed to establish whether or not there was any statistical significance. When the *p* value was less than 0.05, it was considered statistically significant.

### 2.7. Cox Regression Analysis

We used the “survival” package in R to carry out a univariate Cox analysis on the DEGs [27].

### 2.8. Difference Analysis of Scores with Clinical Stages

The data on the clinicopathological characteristics of the ESCC samples that corresponded to them were retrieved from TCGA. The analysis was carried out using the R programming language, and the significance test used was either the Wilcoxon rank sum or the Kruskal-Wallis rank sum test, depending on the number of clinical stages that were being compared.

### 2.9. Immune Infiltration Analysis in ESCC Dataset

We made use of CIBERSORT so that we could investigate the enrichment of immune cells in the tumor microenvironment of ESCC patients. Analyses were performed on the relative abundance of 22 different types of invading immune cells, including T, B, and NK cells, as well as macrophages, for each sample. Spearman's correlation was utilized in order to explore the correlations between essential gene expression and immune cells that were inferred by CIBERSORT. In order to compare the locations of immune cells in groups with high and low levels of gene expression, a Wilcoxon test was carried out.

### 2.10. Statistical Analysis

Utilizing the R programming language, statistical analyses were carried out. A *p* < 0.05 was considered statistically significant.

## 3. Results

### 3.1. Survival Analysis of ESCC Patients in Three Different Scores

**Figure 1:**
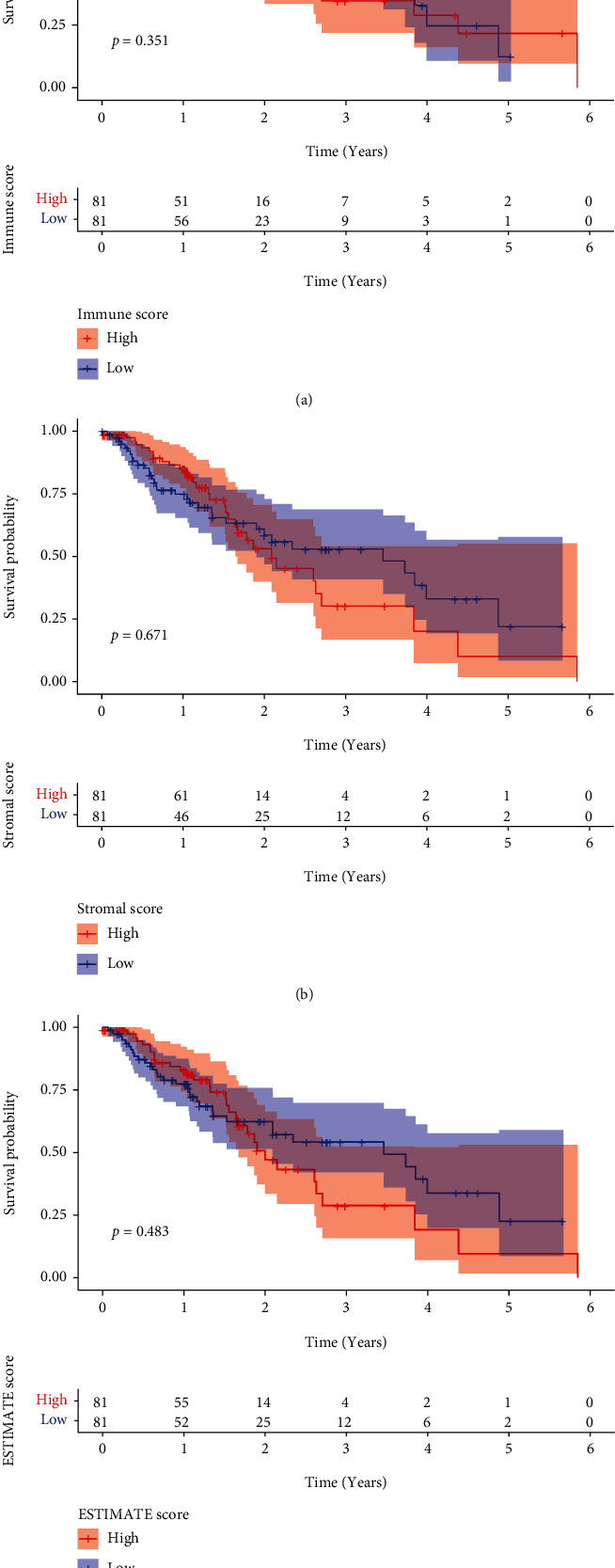
Associations between Immune/Stromal/ESTIMATE scores and survival rates in ESCC patients from TCGA datasets. Kaplan-Meier survival analysis for (a) ImmuneScore, (b) StromalScore, and (c) ESTIMATEScore.

In order to profile the relationship that existed between the various scores and the outcomes of the patients, we employed a combination of ESTIMATE algorithms and Kaplan-Meier survival analyses. ImmuneScore (Figure 1(a)), StromalScore (Figure 1(b)), and ESTIMATEScore (Figure 1(c)) were found to have no correlation with overall survival in ESCC patients.

### 3.2. Analysis of the Correlations between Scores and Clinical Features of Patients with ESCC

**Figure 2:**
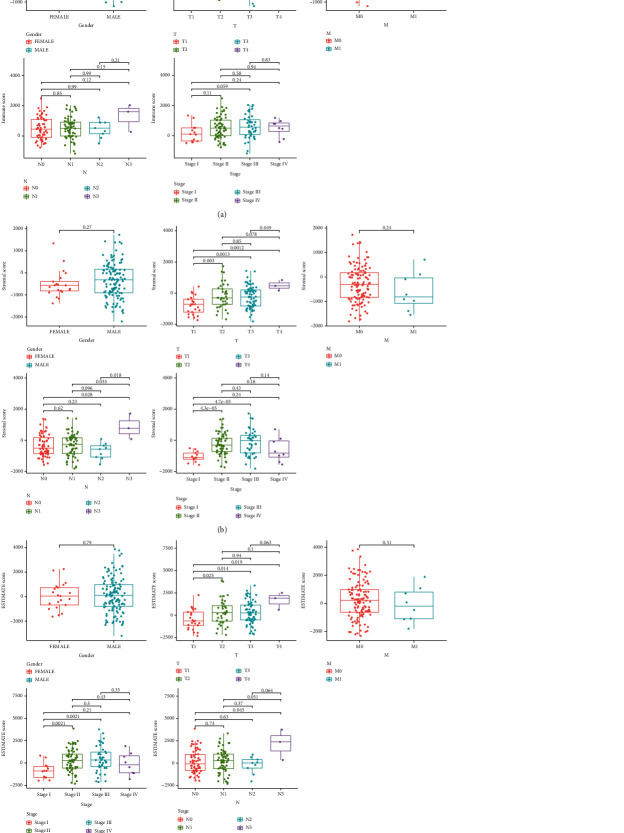
Associations of Immune/Stromal/ESTIMATE scores with clinical factors. (a) ImmuneScore, (b) StromalScore, and (c) ESTIMATEScore.

After that, we performed an analysis to see whether or not there was a correlation between clinical factors of ESCC patients and the scores. We observed that ImmuneScore did not have a significant link with a number of clinical features of ESCC patients, including gender and TMN stage (Figure 2(a)). However, we observed that a higher StromalScore was related to advanced T stages and clinical stages (Figure 2(b)). Mover, we found that higher ESTIMATEScore predicted an advanced T stages and clinical stages (Figure 2(c)).

### 3.3. DEGs Shared by ImmuneScore and StromalScore

**Figure 3:**
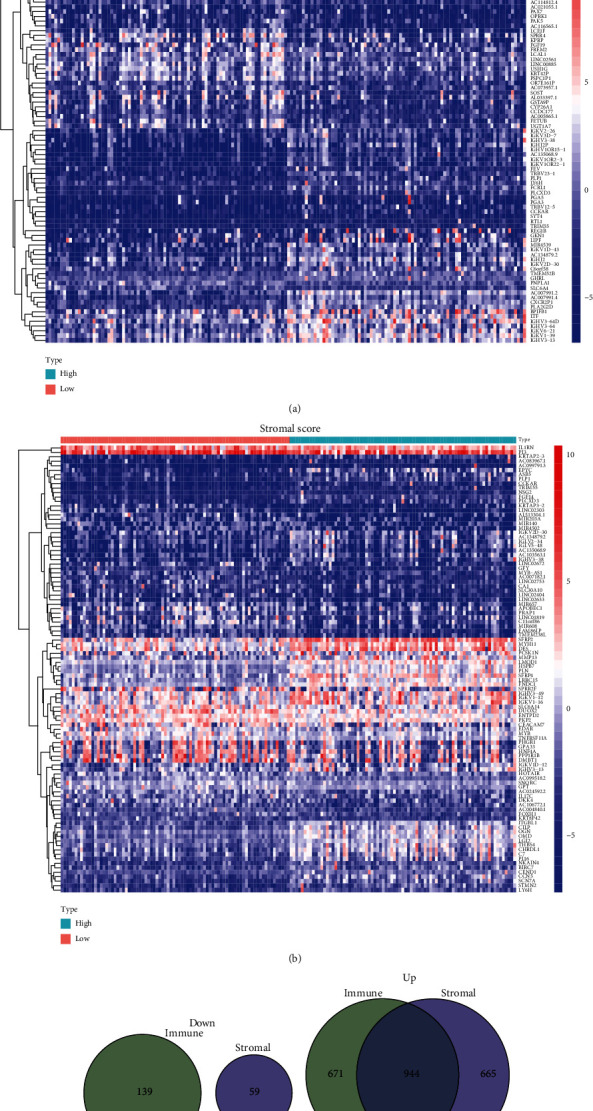
The discovery of DEGs that are common to both the ImmuneScore and the StromalScore. (a) A heatmap for DEGs that was developed by comparing the group with the high score to the group with the low score using ImmuneScore. (b) Heatmap for DEGs in StromalScore. (c, d) Diagrams in the form of Venn plots illustrating upregulated and downregulated DEGs that are common to both ImmuneScore and StromalScore.

The comparative analysis between samples with high scores and those with low scores was carried out in order to determine the precise variations of gene profile in TME relating immunological and stromal components. ImmuneScore provided a total of 1754 DEGs, which are significantly different from the median (samples with high score vs. low score). There were 1615 genes that showed an increase in expression, whereas 139 genes showed a decrease (Figure 3(a)). In a similar fashion, 1668 DEGs were derived using StromalScore. These differentially expressed genes included 1609 genes with an increase in expression and 59 genes with a decrease in expression (Figure 3(b)). The intersection analysis that was presented in the form of a Venn diagram revealed that there was a total of 944 upregulated genes that had the same high score in both the ImmuneScore and the StromalScore and that there was a total of 0 downregulated gene that had the same low score. Both of these scores were determined by the ImmuneScore and the StromalScore (Figures 3(c) and 3(d)). These DEGs could have been the deciding factor in determining the status of the TME.

### 3.4. Functional Correlation Assays

**Figure 4:**
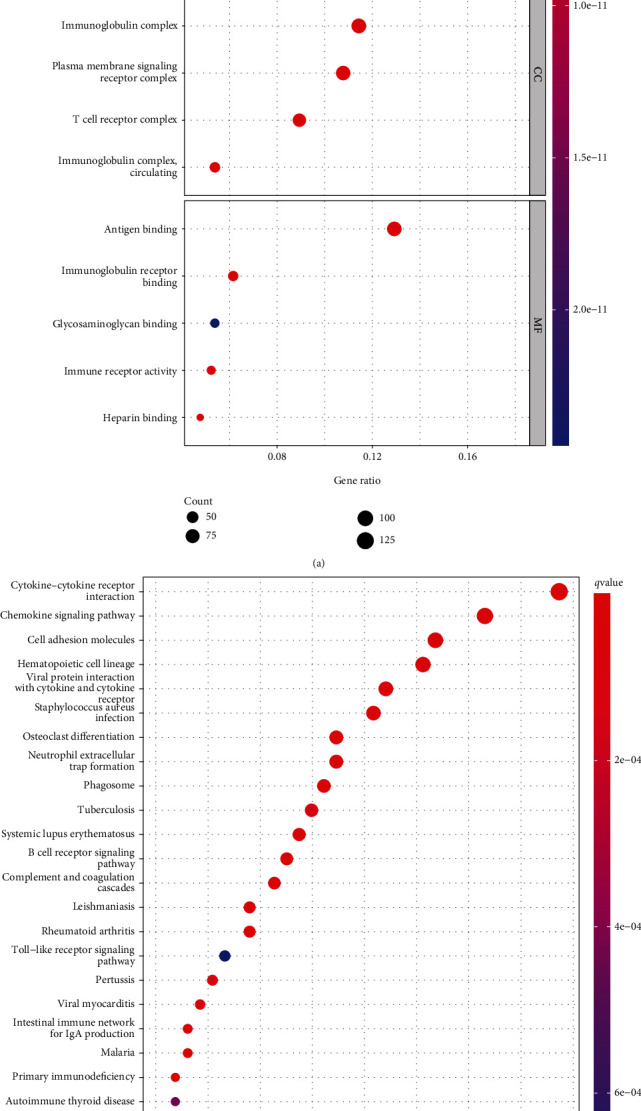
Enrichment analysis of DEGs. (a) GO enrichment analysis. (b) EGG pathway analysis.

Enrichment analyses of GO were carried out in order to learn more about the role of DEGs. The results indicated that the DEGs were mainly related to immune response-activating cell surface receptor signaling pathway, immune response-activating signal transduction, lymphocyte-mediated immunity, external side of plasma membrane, immunoglobulin complex, plasma membrane signaling receptor complex, T cell receptor complex, immune receptor activity, glycosaminoglycan binding, immunoglobulin receptor binding, and antigen binding (Figure 4(a)). The results of KEGG assays revealed that the DEGs were mainly enriched in chemokine signaling pathway, cytokine-cytokine receptor interaction, cell adhesion molecules, osteoclast differentiation, neutrophil extracellular trap formation, phagosome, tuberculosis, and B cell receptor signaling pathway (Figure 4(b)).

### 3.5. The Identification of Survival-Related DEGs in ESCC Patients

**Figure 5:**
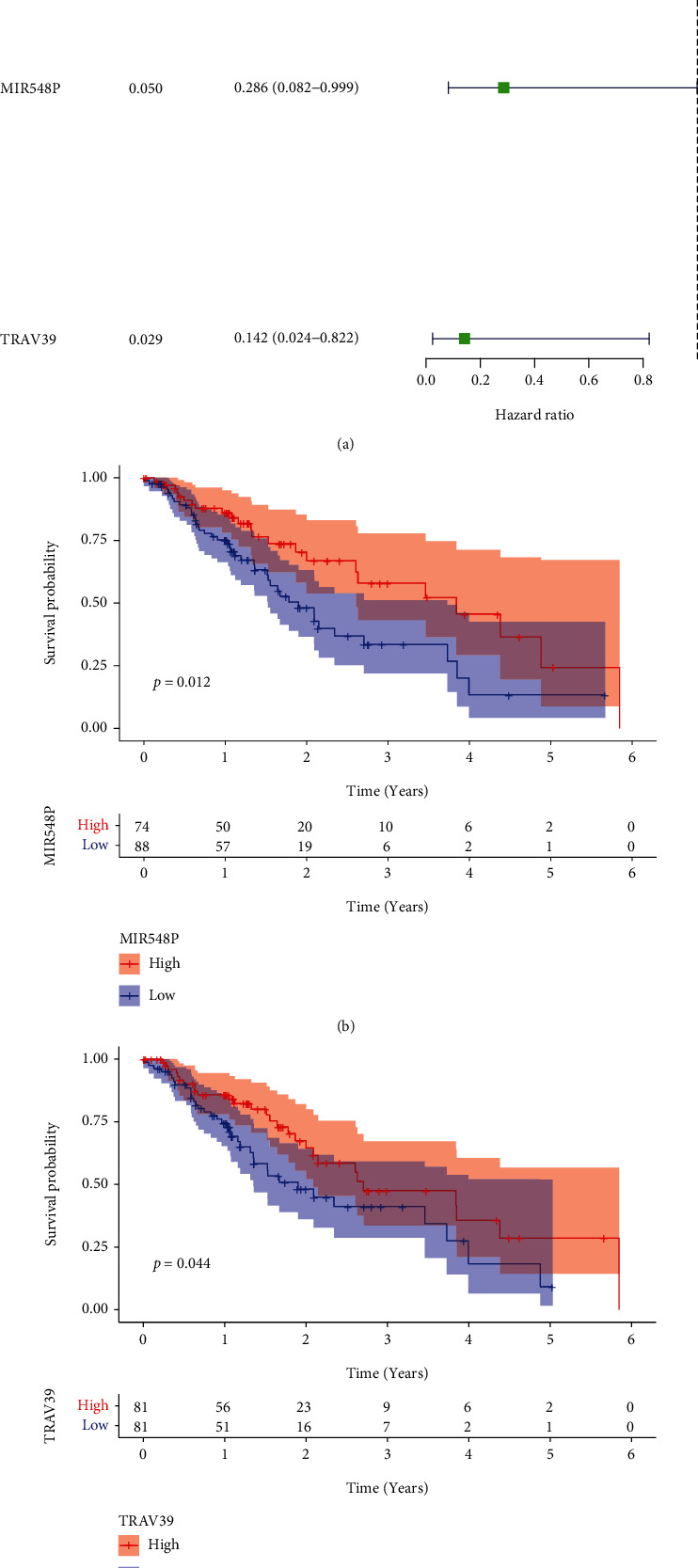
The identification of survival-related DEGs. (a) Univariate analysis was applied to screen the survival-related DEGs in ESCC patients based on TCGA datasets. (b) Kaplan-Meier curves for overall survival after surgery according to expression of MIR548P and TRAV39 expression in ESCC patients.

We carried out a univariate Cox regression on 944 DEGs in order to investigate the crucial genes that play functional roles in ESCC. Only MIR548P and TRAV39, as can be shown in Figure 5(a), were found to be related to an increased likelihood of overall survival among ESCC patients. According to the findings of the Kaplan-Meier method, the 5-year overall survival rate of patients whose MIR548P expression was low was noticeably lower than that of patients whose MIR548P expression was high. This difference was statistically significant (Figure 5(b)). A finding that was quite similar to this one was noticed when patients exhibited a low expression of TRAV39 (Figure 5(c)).

### 3.6. Relationships between MIR548P and TRAV39 Expressions and Clinicopathological Features in ESCC

**Figure 6:**
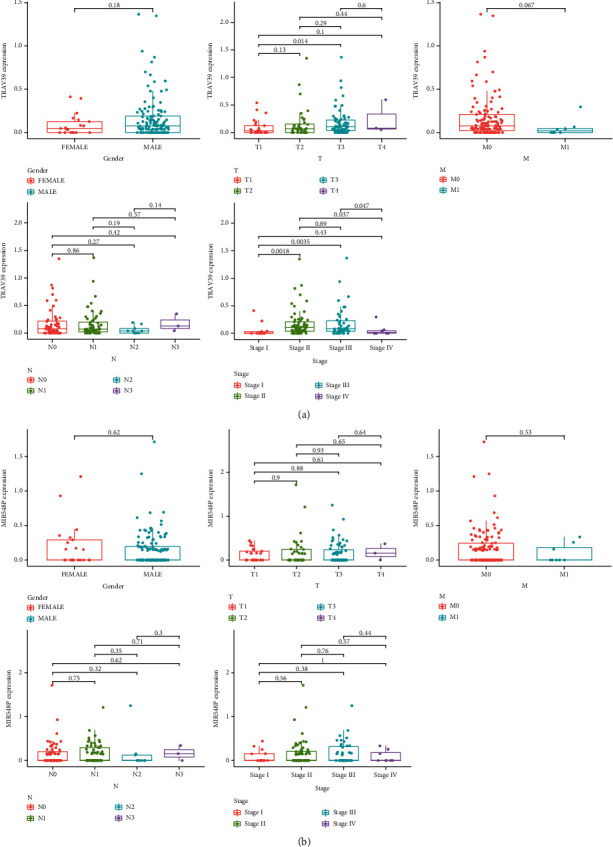
Relationships between (a) MIR548P and (b) TRAV39 expressions and clinicopathological features in ESCC.

In order to investigate the connection between the expressions of MIR548P and TRAV39 and the clinicopathological factors of human ESCC, clinical follow-up information was gathered from all of ESCC patients. Our research revealed that an elevated level of TRAV39 expression was associated with an advanced clinical stage in ESCC patients (Figure 6(a)). On the other hand, we did not discover any data that supported the hypothesis that there was a positive link between the expression of MIR548P and the clinicopathological characteristics of ESCC patients (Figure 6(b)).

### 3.7. Correlation of MIR548P and TRAV39 with the Proportion of TICs

**Figure 7:**
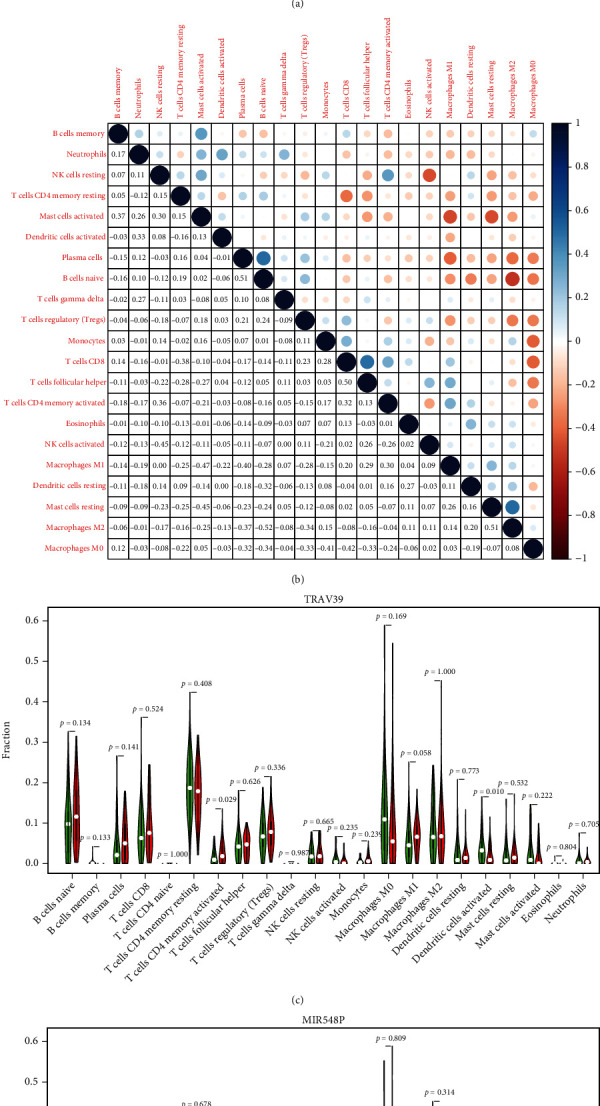
TIC profile in the tissue samples from the tumors and correlation analysis. (a) The distribution of the 21 different types of TICs found in ESCC tumor samples is shown as a barplot. (b) A heatmap displaying the correlation between 21 different types of TICs, with a number in each little box reflecting the *p* value of correlation between two different types of cells. (c, d) All ESCC cases were divided into the high and low (c) MIR548P and (d) TRAV39 expression groups, based on the median of MIR548P and TRAV39 expressions, and the Wilcoxon rank-sum test was carried out.

**Figure 8:**
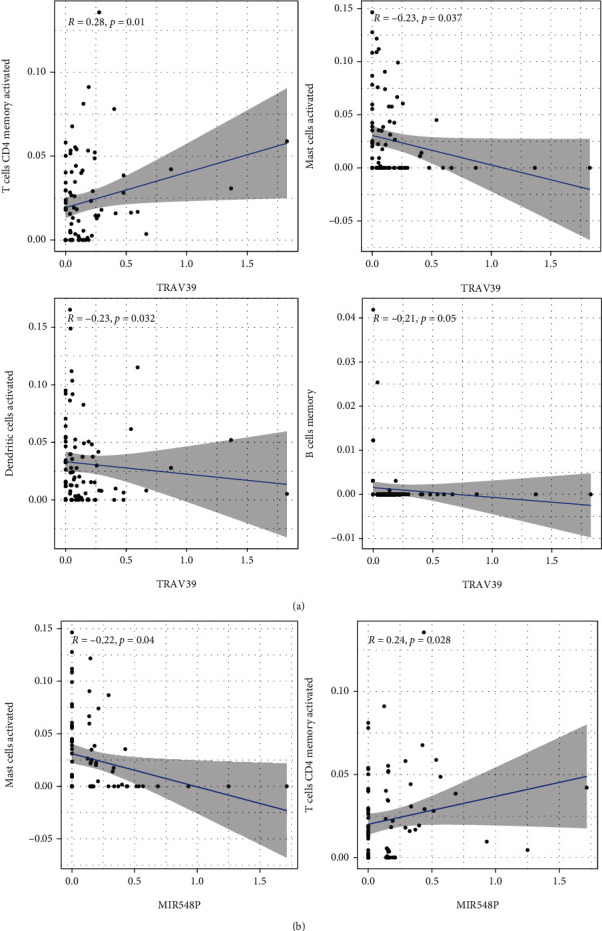
Correlation of TIC proportion with the expression of (a) TRAV39 and (b) MIR548P.

In order to provide additional evidence that MIR548P and TRAV39 expressions were correlated with the immune microenvironment, it was determined with the use of the CIBERSORT algorithm what proportion of immune subsets had invaded the tumor, and 21 different immune cell profiles were constructed using ESCC samples (Figures 7(a) and 7(b)). The dysregulated levels of immune cells are shown in Figures 7(c) and 7(d). Importantly, we found that TRAV39 expression was positively associated with T cell CD4 memory activated while negatively associated with B cell memory, dendritic cells activated, and mast cells activated (Figure 8(a)). In addition, we found that MIR548P expression was negatively associated with mast cells activated while positively associated with T cell CD4 memory activated (Figure 8(b)). Thus, our findings suggested MIR548P and TRAV39 were involved in the function of immune microenvironment.

## 4. Discussion

ESCC is one of the most common forms of aggressive cancer worldwide, and it is especially prevalent in China, where it ranks as the fourth most common reason for people to pass away from cancer-related causes [28, 29]. The 5-year survival rate for ESCC patients is roughly 17%. Due to the absence of a single, reliable clinical approach for early identification, ESCC is associated with a typically dismal prognosis globally [30, 31]. ESCC accounts for approximately 90% of all occurrences of esophageal cancer. The ongoing dismal clinical outcome suggested that there was an immediate need to increase our understanding of the molecular mechanism behind the carcinogenesis of ESCC [32, 33]. The above knowledge could help in the creation of innovative ways for predicting the patient's prognosis. There was an increasing body of evidence suggesting that aberrant regulation of certain proteins was essential for the advancement of ESCC [34, 35]. Therefore, the mortality rate of ESCC patients can be lowered and clinical outcomes can be improved by the discovery of novel biomarkers that can be used in early diagnosis and prognostic assessment to better personalize therapy.

The TME was home to a wide variety of cell types, all of which are integral parts of tumor tissues and play a crucial part in both the beginning and progression of cancers [36]. The cells and substances that make up the TME were constantly undergoing change, which served to both identify characteristics of the tumor and encourage immune evasion, growth, and metastasis [37]. Multiple studies have shed light on the therapeutic relevance of the TME in the prediction of therapy efficacy and patient prognosis [38]. In recent years, various medications that target the TME, such as immune checkpoint inhibitors and angiogenesis inhibitors, have shown significant success in regulating the progression and spread of malignancies [39, 40]. These drugs included angiogenesis inhibitors. In this study, we determined the percentages of TME components and carried out survival analysis pertaining to those findings. However, according to the findings, neither the ESTIMATEScore nor the StromalScore nor the ImmuneScore was substantially connected to the overall survival rate of patients with ESCC. The immunological state was shown to be connected with the clinical outcome of ESCC patients, which was not consistent with our data but has been corroborated by an increasing number of research. I hypothesized that the small number of participants could be responsible for this outcome. Then, DEGs were discovered by TME score-related gene expression difference analysis, and GO and KEGG enrichment analyses were carried out. The results illustrated that the DEGs were enriched in cytokine-cytokine receptor interaction, chemokine signaling pathway, cell adhesion molecules, osteoclast differentiation, neutrophil extracellular trap formation, phagosome, tuberculosis, and B cell receptor signaling pathway. The univariate Cox regression analysis was also performed using the DEGs. Only MIR548P and TRAV39 were related with an increased likelihood of overall survival in ESCC patients, according to our findings. In addition, survival experiments demonstrated that a high expression of MIR548P and TRAV39 predicted a decreased overall survival rate. Moreover, we found that TRAV39 was positively associated with an advanced clinical stage. It has been known to us that clinical stage is a very important index to predict the prognosis of patients. In clinical practice, doctors also make different treatment plans according to the clinical stages. Our findings highlighted the important roles of TRAV39 expression in ESCC progression. To date, the expression and function of MIR548P and TRAV39 were rarely reported.

Immune cells have the ability to mediate chemotherapeutic resistance and sensitivity, which can increase patients' chances of survival when they have ESCC [14]. It has been established beyond a reasonable doubt that the primary immune cell subtypes that are positively associated to the important genes include immune effector cells (M1 macrophages and CD8 T cells), plasma cells that have the capacity to secrete antibodies, Treg cells, and activated memory CD4 T cells [41, 42]. Immune cells such as naive CD4 T cells and M0 macrophages, activated DC cells, and memory B cells are examples of immune cells that have a negative relationship with important genes. One of the hallmark host immunological responses to tumor cells is the infiltration of immune cells, which has been linked in numerous studies to both the initiation and progression of cancers. This reaction is one of the hallmarks of the immune system of the host. It has been observed that a high expression of Tregs and a low ratio of M0 macrophages are two factors that lead to a positive prognosis of overall survival and disease-free survival in patients with ESCC [43, 44]. It is commonly accepted that CD8+ T lymphocytes destroy tumor cells by attaching to MHCI antigens. Additionally, the total number of CD8+ cells has been shown to have a favorable correlation with tumor grade and a better patient prognosis in cases of ESCC [14, 43]. Memory CD4+ T cells, meantime, suppress the expansion of tumor cells by encouraging the multiplication of CD8+ cells. The anticancer activity of memory CD4+ T cells is further supported by previous findings demonstrating that an increase in disease-free survival of ESCC patients is directly associated to an increase in activated memory CD4+ T cells [44, 45]. In this study, we found that TRAV39 expression was positively associated with T cell CD4 memory activated while negatively associated with B cell memory, dendritic cells activated, and mast cells activated. In addition, we found that MIR548P expression was negatively associated with mast cells activated while positively associated with T cell CD4 memory activated. Our findings suggested that high expression of TRAV39 and MIR548P predicted a poor prognosis due to the promotion of antitumor immunity in ESCC.

## 5. Conclusion

Our findings identified two novel regulators involved in ESCC progression. The expressions of TRAV39 and MIR548P might aid in the prediction of the clinical outcome of ESCC patients, especially the status of TME. TRAV39 and MIR548P can be utilized as a promising modulator in the development of immunotherapy for ESCC.

## Data Availability

The authors confirm that the data supporting the findings of this study are available within the article.
